# Liver Resections Combined with Closure of Loop Ileostomies: A Retrospective Analysis

**DOI:** 10.1155/2008/501397

**Published:** 2008-12-10

**Authors:** Jeffrey T. Lordan, Angela T. Riga, Nariman D. Karanjia

**Affiliations:** Regional Hepato-Pancreatico-Biliary Unit for Surrey and Sussex, The Royal Surrey County Hospital, Egerton Road, Guildford, Surrey GU2 7XX, UK

## Abstract

*Background*. The management of patients with colorectal liver metastases and loop ileostomies remains controversial. This study was performed to assess the outcome of combined liver resection and loop ileostomy closure. *Methods*. Analysis of prospectively collected perioperative data, including morbidity and mortality, of 283 consecutive hepatectomies for colorectal liver metastases was undertaken. Consecutive liver resections were performed from 1996 to 2006 in one centre by a single surgeon (NDK). Fourteen of these patients had combined liver resection and ileostomy closure. Case-matched analysis was undertaken. *Results*. Six (2.2%) patients died in the hepatectomy only group and none died in the combined group. There was no difference in operative blood loss between the two groups (0.09). Perioperative morbidity was 36% in the combined group and 23% in the hepatectomy alone group (*P* = 0.33). Mean hospital stay was 14 days in the combined group and 11 days in the hepatectomy only group (*P* = 0.046). Case-matched analysis showed a significant increase in hospital stay (*P* = 0.03) and complications (*P* = 0.049) in the combined group. *Conclusion*. In patients with CRLM, combined liver resection and closure of ileostomy may be associated with a higher operative morbidity and a prolonged hospital stay.

## 1. INTRODUCTION

 Colorectal cancer is the second leading cause of cancer death in the Western world [1, 2]. A quarter of the patients with colorectal cancer have liver metastases at presentation and 40% go on to develop liver metastases at a later stage [3, 4]. Liver resection remains the only potentially curative treatment for colorectal liver metastases (CRLM) [2–4]. Liver resections taking place in specialised high-volume centres are well-tolerated procedures with low morbidity and mortality.

A number of patients with low-rectal cancers undergo anterior resection with defunctioning loop ileostomy [5]. The closure of loop ileostomies is a reasonably straightforward procedure with low mortality and morbidity and short hospital stay [5].

The aim of the study was to look at the impact of concomitant loop ileostomy closures on the perioperative morbidity and length of hospital stay after a liver resection.

## 2. MATERIALS AND METHODS

An analysis of 283 consecutive hepatic resections for CRLM, undertaken between September 1996 and August 2006, was performed. Data were collected and analysed prospectively by a dedicated whole-time data clerk.

All patients had a staging laparoscopy and intraoperative ultrasonography. Postoperative analgesia was achieved with thoracic epidural.

Perioperative mortality was defined as death during the same hospital admission or within 30 days of the date of the operation if the patient was discharged earlier. Postoperative complications were classified as cardiac, respiratory, renal, hepatic impairment, thromboembolic, sepsis, and wound infections.

All loop ileostomies were closed using interrupted sutures. In patients who underwent concomitant liver resection and ileostomy closure, no other procedures were performed.

Due to the discrepancy in the number of patients between the two groups, a case-matched analysis was carried out. This directly compared the patients who underwent concomitant loop ileostomy closure and liver resection versus an equal number of patients who underwent liver resection alone. The patients who underwent liver resection alone were matched for type of liver resection, age, and American Society of Anesthesiologists (ASA). They also had loop ileostomies that had been formed at their primary colorectal resection which were reversed at a separate time to the liver resection.

Chi-squared and *t*-tests were used to analyse data. *P*-value less than .05 was considered to be significant.

## 3. RESULTS

Of the 283 patients undergoing liver resection for CRLM, 14 had a combined liver resection and concomitant loop ileostomy closure. The median time from the formation of loop ileostomy to closure was 252 days (range 68 to 662 days).

Table 1 demonstrates patients’ demographics and perioperative outcomes. There was no significant difference in age or median blood loss between the two groups (*P* = .13 and .09, resp.). Hospital stay was longer in the group who had a concomitant liver resection and loop ileostomy closure (*P* = .04). There was no significant difference in maximum tumour size (*P* = .16). Hospital stay was significantly longer in the combined group (13 days versus 10 days, *P* = .046). The number of repeat resections in the hepatectomy alone group was significantly higher (*P* < .0001).

Table 2 shows the number and type of perioperative complications. There were 62 (23%) complications in the group who had a liver resection alone compared with 5 (36%) complications in the concomitant group (*P* = .33).

However, the number of serious complications (grade III or above, according to the classification of surgical complications [6]) in the group who had a liver resection alone was 18 (6.7%) versus 2 (14%) in the concomitant group (*P* = .42).

There were 6 (2.2%) postoperative deaths in the hepatectomy group. 3 died due to hepatic insufficiency, 2 due to cardiac complications, and 1 due to sepsis. There were no deaths in the group who had a liver resection and loop ileostomy closure.

Table 3 demonstrates a case-matched analysis of an equal number of patients to the group who had concomitant loop ileostomy closure and liver resection. There was no difference in age, type of liver resection, ASA, number and distribution of liver metastases, maximum tumour size, or blood loss. Hospital stay was significantly longer in the concomitant group (*P* = .03) as was the complication rate (*P* = .049), although serious complication rates were not significantly different (0.13). There were no postoperative deaths in these two groups.

## 4. DISCUSSION

Loop, or defunctioning, ileostomies are often created to minimise the impact of peritoneal sepsis from an anastomotic dehiscence following coloanal or low-colorectal anastomosis [5, 7]. However, it probably does not reduce the incidence of anastomotic leak [5, 8–10]. The patients in this series appear to have had a substantial delay in time from formation to closure compared to the literature [5, 7]. Loop ileostomy closure is often considered low priority by clinicians [5, 7], and it is likely that more consideration was given to treating the liver metastases, with neoadjuvant chemotherapy followed by liver resection.

Patients suitable for hepatectomy often request a closure of their loop ileostomy at the time of liver resection. However, to the authors’ knowledge, there is no documented evidence demonstrating the safety of this combined procedure compared with hepatectomy alone.

Anecdotally, it was felt in our institution that loop ileostomy closure combined with liver resection increased morbidity. The analysis of the data shows that there was a substantial increase in complications with the combined procedure, although it did not reach significance, possibly due to the low numbers involved. Although there were no postoperative deaths in the group who had the combined procedure, there is an evidence that increased frequency of complications during the perioperative period can be associated with a significantly higher mortality regarding hepatectomy [11]. The analysis of the case-matched series, however, did show a significant increase in complications in the concomitant group, although there was no difference in serious complications [6]. Further evidence of the impact of combining these two procedures was demonstrated by the significant increase in hospital stay both in the overall analysis and the case-matched analysis.

The literature reports perioperative morbidity regarding liver resection for CRLM at 13–37% [1, 12, 13]. However, complication rates associated with hepatectomy have steadily improved over the years partly due to accurate patient assessment and selection and improved critical care. The mortality in the hepatectomy alone group was 2.2%. In the literature, operative mortality for liver resection has reduced over the years to less than 5% in experienced centres due to improved patient assessment and selection [14, 15]. Three of the six patients in our series died due to hepatic insufficiency. This may be related to intraoperative Pringle manoeuvres, or the use of neoadjuvant chemotherapy, which can be associated with nonalcoholic steatohepatitis (NASH) [16].

Recently, articles have reported that loop stoma closure as a procedure in its own right can be associated with a substantial morbidity, and is not necessarily the “easy, low-risk” procedure it may once have been considered [17, 18]. This may explain the substantial increase in morbidity in patients undergoing closure of loop ileostomy combined with hepatectomy found in this study. The increased complication rate may also explain the significantly higher hospital stay seen in the group who had the combined procedure.

Although there were differences in outcomes between the two groups, the study is limited not only due to the numbers but also due to the potential difference in disease biology between them. However, a recent study comparing hepatectomy and synchronous colonic resection versus colonic resection alone concluded that combining the procedure significantly increases the hospital stay and morbidity rate, reporting a median hospital stay of 13.9 days and complication rate of 56.3% [19].

Patients occasionally request that their loop stoma be closed at the time of hepatectomy. In such cases, the risks demonstrated in this article can be presented to patients before making an informed decision.

In conclusion, combined liver resection and concomitant closure of loop ileostomy may be associated with increased perioperative morbidity and prolonged hospital stay.

## Figures and Tables

**Table 1:** Patients’ demographics and operative outcomes.

	Hepatic resection alone (*n* = 269)	Hepatic resection + loop stoma closure (*n* = 14)	*P*-value
Male:female	2.2:1	1.8:1	N/A
Mean age/years (range)	66.5 (33 to 85.4)	62 (42 to 73)	.13

ASA			N/A
(i) 1	51 (19%)	5 (36%)	
(ii) 2	148 (55%)	6 (43%)	
(iii) 3/4	36 (13%)	0	
(iv) Not recorded	34 (13%)	3 (21%)	

Type of liver resection			N/A
(i) Hemihepatectomy	95 (35%)	6 (43%)	
(ii) Extended hemihepatectomy	67 (25%)	4 (29%)	
(iii) Parenchymal sparing resection	107 (40%)	4 (29%)	

Number and distribution of liver metastases			N/A
(i) 1	125	8	
(ii) 2	61	2	
(iii) 3	33	3	
(iv) 4	16	1	
(v) >4	22	0	
(vi) Bilateral liver mets	54	0	
(vii) Complete response to chemotherapy	12	0	

Repeat resections	12	0	< .0001
Maximum tumours size/mm	31.5 (3 to 165)	28.5 (5 to 85)	.16
Mean blood loss/mL (range)	262 (0 to 2500)	219.2 (0 to 2100)	.09
Mean hospital stay/days (range)	11 (3 to 53)	14 (8 to 23)	.046

ASA = American Society of Anesthesiologists.

**Table 2:** Perioperative complications.

Complication	Hepatic resection alone (*n* = 269)	Hepatic resection + loop stoma closure (*n* = 14)
Cardiac	14	0
Respiratory chest infection	7	1
Respiratory failure	7	0
Renal	3	0
Liver insufficiency	5	0
Thromboembolic	1	0
Sepsis	5	0
Intestinal obstruction	0	1
Deep wound infection	9	2
Bile leak	11	1
Total (%)	62 (23%)	5 (36%)

*P* = .33.

**Table 3:** Case-matched analysis.

	Hepatic resection alone (*n* = 14)	Hepatic resection + loop stoma closure (*n* = 14)	*P*-value
Male:female	2.6:1	1.8:1	N/A
Mean age/years (range)	59.7 (34 to 77)	62 (42 to 73)	.6

ASA			N/A
(i) 1	5 (36%)	5 (36%)	
(ii) 2	6 (43%)	6 (43%)	
(iii) 3/4	2 (14%)	0	
(iv) Not recorded	1 (7%)	3 (21%)	

Type of liver resection			N/A
(i) Hemihepatectomy	6 (43%)	6 (43%)	
(ii) Extended hemihepatectomy	3 (21%)	4 (29%)	
(iii) Parenchyma sparing resection	5 (36%)	4 (29%)	

Number and distribution of liver metastases			N/A
(i) 1	8	8	
(ii) 2	4	2	
(iii) 3	1	3	
(iv) 4	0	1	
(v) >4	1	0	
(vi) Bilateral liver mets	3	0	

Maximum tumours size/mm	26.5 (4 to 130)	28.5 (5 to 85)	.5
Mean blood loss/mL (range)	216 (30 to 2500)	219 (<20 to 2100)	.15
Mean hospital stay/days (range)	11 (6 to 15)	14 (8 to 23)	.03
Complications	1 (7.1%)	5 (36%)	.049
Serious complications (grade III or above) [6]	0	2 (14%)	.13
